# A survey of breastfeeding among women with previous surgery for benign breast disease: a descriptive exploratory study

**DOI:** 10.1186/s13006-024-00647-8

**Published:** 2024-06-05

**Authors:** Siying Mao, Jiafa He, Lezhen Huang, Yang Sun, Yan Dai, Qianqian Guo, Chang Qiu, Xue Song, Xiaojie Lin, Shengying Chen, Lingling Ye, Rui Xu

**Affiliations:** Breast Department, Guangdong Provincial Hospital of Chinese Medicine, The Second Affiliated Hospital of Guangzhou University of Chinese Medicine, Guangzhou, Guangdong China

**Keywords:** Benign breast disease, Mammary lumpectomy, Breast surgery, Breastfeeding, Lactation function

## Abstract

**Background:**

Surgery is the primary treatment for benign breast disease and causes some disruption to the normal physiology of the breast, even when this disruption is localised, it remains unclear whether it affects women’s ability to breastfeed. There are only a few studies describing the experience of breastfeeding in women who have undergone benign breast disease (BBD) surgery.

**Methods:**

We retrospectively analysed data from patients aged 20–40 years in Guangdong, China, who underwent breast lumpectomy for BBD in our department between 01 January 2013 and 30 June 2019, with a follow-up date of 01 February 2022. Patients were included who had a history of childbirth between the time of surgery and the follow-up date. By collecting general information about this group of patients and information about breastfeeding after surgery, we described the breastfeeding outcomes of women of a fertile age who had previously undergone surgery for benign breast disease.

**Results:**

With a median follow-up of 5.9 years, a total of 333 patients met the inclusion criteria. From the breastfeeding data of the first child born postoperatively, the mean duration of ‘exclusive breastfeeding’ was 5.1 months, and the mean duration of ‘any breastfeeding’ was 8.8 months. The rate of ‘ever breastfeeding’ is 91.0%, which is lower than the national average of 93.7%, while the exclusive breastfeeding rate at six months was 40.8%, was higher than the 29.2% national average. The any breastfeeding rate at 12 months was 30.0%, which was well below the 66.5% national average. The common reason for early breastfeeding cessation was insufficient breast milk. A total of 29.0% of patients who had ever breastfed after surgery voluntarily reduced the frequency and duration of breastfeeding on the operated breast because of the surgery.

**Conclusions:**

There are some impacts of BBD surgery on breastfeeding and some may be psychological. Institutions should provide more facilities for mothers who have undergone breast surgery to help them breastfeed, such as conducting community education on breastfeeding after breast surgery, training professional postoperative lactation consultants in hospitals, and extending maternity leave. Families should encourage mothers to breastfeed with both breasts instead of only the non-operated breast.

## Background

Breastfeeding is the best source of nutrition for infants. It is important for short and long-term health of the infant [1]. The World Health Organization (WHO) recommends that breastfeeding should be initiated within one hour of birth and that children should be exclusively breastfed, i.e., offered no other liquids or food, for the first six months of life. From six months onwards, children should start adequate and safe complementary foods while continuing to breastfeed until two years of age or older [2]. The current situation of breastfeeding in China is not encouraging. According to research, the rate of exclusive breastfeeding for infants aged 0–6 months in China has been on a downward trend with the increase in socioeconomic status [3].

Breast disease can affect breastfeeding. Although breast cancer is currently the number one cancer worldwide [4], its incidence is much lower than that of benign breast disease. Benign breast disease (BBD) is very common in women of childbearing age. The most common BBDs are breast hyperplasia and fibroadenomas, which grow abnormally during reproductive years, mainly due to oestrogen exposure, and the development of these diseases is influenced by age, pregnancy and hormonal status [5]. Studies have shown that proliferative BBD is likely to increases the risk of breast cancer in women [6–8]. BBD includes a range of pathological changes, of which approximately 30% involve proliferative benign lesions and 65% include nonproliferative benign lesions. Proliferative BBD with atypical hyperplasia accounts for approximately 3–4% of BBD diagnoses and is associated with a 4-5-fold increased risk of breast cancer [9–11]. Due to the fear of breast cancer, many patients with benign breast disease choose to undergo surgical treatment, which includes breast biopsy and minimally invasive surgery, as well as traditional open surgery.

A report published in 2019 points out that the rate of ever breastfeeding in China is 93.7% [12], and the rate of any breastfeeding at 12 months is 66.5%. In addition, the rate of exclusive breastfeeding for six months in China is 29.2%, which means that no other food or fluid, including water, is provided since birth. This is lower than the world average of 43% and the average of 37% in low- and middle-income countries [12]. Among these countries, the highest rate of exclusive breastfeeding of 36% is found in large cities, while the lowest rate of only 23% is found in small and medium-sized cities. However, the WHO goal is to increase the exclusive breastfeeding rate for the first six months to at least 50% by 2025 [13]. This suggests that we still have a long way to go before reaching the WHO target. In recent years, an increasing number of women have become concerned about the impact of breast surgery on breastfeeding. Only a handful of studies on breast-conserving surgery for breast cancer or breast reduction and reconstructive surgery have explored this issue [14, 15]. The largest number of breast surgeries in China today are for benign breast disease. Therefore, it is very important to find out the breastfeeding status of this large special group of people who have undergone benign breast disease surgery.

In our clinical work, we come into contact and communicate with many young women who suffer from high-risk benign breast diseases, such as atypical ductal hyperplasia and fibroadenoma, and who require surgical treatment. We have found that the most important concerns of these young women of childbearing age are whether they can breastfeed if they undergo BBD surgery. Worry and anxiety are potential risk factors for decreased breastfeeding rates among women, so we need to provide them with real-world data that would eliminate their fears and anxieties. Therefore, we conducted this retrospective survey to describe and analyse real-world breastfeeding outcomes among women of childbearing age who have undergone surgery for benign breast disease. Also to gain a preliminary understanding of the difficulties they encountered during breastfeeding, which will help to disseminate accurate knowledge about breastfeeding to this special population.

## Methods

### Sample and setting

In this study, we retrospectively analysed patients in Guangdong, China, who underwent mammary lumpectomy for BBD in our department from 01 January 2013 to 30 June 2019 and met the following criteria: (1) were 20–40 years of age; (2) had BBD confirmed by postoperative paraffin pathology, including fibroadenoma, fibrocystic hyperplasia of the breast, papilloma, benign phyllode tumour, atypical ductal hyperplasia, atypical lobular hyperplasia, etc.; (3) had a history of childbirth between the time of surgery and the follow-up date; (4) had no personal psychiatric history; (5) had no other related conditions that could affect breastfeeding; (6) had no history of breast malignancy; and (7) had no serious life-threatening illnesses. Our study began with the follow-up of patients on 01 February 2022, with a median follow-up of 5.9 years, a total of 333 patients met the inclusion criteria.

### Data collection

Data were collected through telephone interviews, and the review of clinical case information and outpatient consultations. We collected clinical information from the enrolled patients, including general information (age, height, weight, body mass index [BMI], education level), history of previous major surgery, information on birth history (age at menarche, age at first birth, parity) and information on breast surgery and pathology (method of surgery, location of surgical incision, number of tumours removed, size and exact location of tumours removed, postoperative paraffin pathology). The self-report questionnaire for telephone follow-up was used to collect information on breastfeeding, including the type of breastfeeding of the first child born after surgery (exclusive breastfeeding or mixed breastfeeding), the duration of breastfeeding, the reasons for the cessation of breastfeeding, and whether there was any mastitis on the operated breast. The specific questionnaire content is detailed in Table 1.

**Table 1:** Questionnaire on postoperative breastfeeding

Some of the key questions
1. How many children have been born since the operation?
2. What was the exact timing of postoperative fertility?
3. Was breastfeeding ever performed after surgery?
4. Duration of exclusive breastfeeding for the first child born postoperatively?
5. Duration of any breastfeeding for the first child born postoperatively?
6. What were the reasons for stopping breastfeeding?
7. Was there a difference in breastfeeding between the two breasts? If so, which breast produced more milk?
8. Have you ever taken the initiative to reduce the frequency and duration of breastfeeding on the operated breast because of the surgery?
9. Has the operated breast experienced redness, swelling and pain during breastfeeding?

Our study defined exclusive breastfeeding and any breastfeeding strictly according to WHO standards. Exclusive breastfeeding was defined as no other food or fluids, including water, being offered since birth. Any breastfeeding meant that while continuing to breastfeed, children could start eating safe and adequate complementary foods. Ever breastfeeding is defined as the ability of the mother to breastfeed for not less than one day after delivery, including exclusive breastfeeding or any breastfeeding. The rate of ever breastfeeding is the proportion of patients who were able to breastfeed for not less than one day after delivery as a percentage of all enrolled patients. Early cessation of breastfeeding is defined as breastfeeding for less than 15 days, including exclusive breastfeeding or any breastfeeding. The average time taken to complete the telephone questionnaire was 15 min.

### Surgical details

All included patients with BBD under went breast lumpectomy. The incision for traditional open surgery is usually chosen as a curved incision along the areola or a surface incision of the mass. In the case of a curved areolar incision, we would carefully detach the flap under the skin and radially incise the gland after reaching the margin of the mass and remove the breast lesion completely. With a superficial incision, we would also radially incise the gland at the margin of the mass and remove the breast lesion completely. During the procedure, the breast tissue behind the nipple areola would be protected, avoiding damage to the ducts behind the nipple areola and leaving most of the breast lobules and their lactiferous ducts intact. Minimally invasive surgery refers to the complete excision of breast lesions using the Mammotome (vacuum-assisted breast biopsy device) biopsy system [16, 17].

### Description of results and statistical analysis

We used a questionnaire to collect information about breastfeeding from patients. Breastfeeding duration was displayed graphically using survival plots, with age of the infant on the horizontal axis and proportion of infants being breastfed on the vertical axis.

Studies have suggested that breastfeeding is affected by income level and urbanisation [18, 19], and that China’s first-tier cities are significantly better than non-first-tier cities in terms of economy, education, healthcare and public facilities. To reduce the influence of economic factors, all patients were analysed and compared in two different groups, according to whether they lived in a first-tier city or not. In addition, it has also been reported that parity affects breastfeeding [20]. In order to reduce this effect, we analysed and compared all patients in two different groups according to whether they had a history of childbirth prior to surgery.

Data were analysed using SPSS, ver. 24, considering *P* < 0.05 as significant. The mean ± SD (standard deviation) and percentage distributions are used for the statistical description of general information and clinically relevant characteristics of the enrolled patients. The data were verified for normality using the quantile‒quantile plot and the Shapiro‒Wilk test. For continuous variables with a normal distribution, independent sample t tests and one-way ANOVA were used to compare means between groups and in other cases. For parameters with nonnormal distributions, the Mann‒Whitney U test was used to compare unpaired samples. Categorical variables were compared using the χ2 test and Fisher’s exact test.

## Results

### Sociodemographic and general clinical information

Our study centre is located in Guangzhou, Guangdong Province, China. A large proportion of the patients included lived in the Pearl River Delta region of Guangdong Province, a more economically developed area. The mean age of the 333 patients enrolled was 28.0 years, the mean diameter of the largest tumour removed was 2.4 cm, the mean number of tumours removed was 1.5, the mean age at first birth was 28.7 years, and the mean interval between surgery and first postoperative breastfeeding was 2.8 years. A total of 86.5% of the patients had a college education or above, 10.2% had below a college education, and 30.6% had a history of childbirth prior to surgery.

Eighty-eight-point 1% of patients underwent surgery on one breast, 11.9% underwent surgery on both breasts, 21.0% underwent minimally invasive surgery, 78.4% underwent traditional open surgery. Seventy-point-9% had a curved areolar incision, 24.0% had a surface incision of the mass, 76.0% had a postoperative pathology result of simple fibroadenoma and 81.7% did not experience redness, swelling or pain in the operated breast during breastfeeding (Table 2).

**Table 2:** Baseline characteristics of enrolled patients grouped by history of childbirth prior to surgery

	Overall (333) ‡	Patients with no history of childbirth prior to surgery (231) ‡	Patients with a history of childbirth prior to surgery (102) ‡	*P* value (t/U/χ2 test)
Age (years)	28.01 (4.11)	26.41 (3.28)	31.64 (3.44)	0.000*
Height (m)	1.59 (0.05)	1.58 (0.05)	1.60 (0.05)	0.016*
Weight (kg)	50.08 (6.46)	49.14 (6.12)	52.21 (6.72)	0.000*
BMI	19.88 (2.26)	19.63 (2.18)	20.46 (2.35)	0.002*
Number of tumours removed	1.53 (0.95)	1.59 (1.02)	1.39(0.73)	0.048*
Diameter of the largest tumour (cm)	2.36 (1.04)	2.47 (1.06)	2.11 (0.97)	0.004*
Age at first birth (years)	28.73 (3.47)	29.78 (3.47)	26.35 (3.39)	0.000*
Interval between surgery and the first postoperative breastfeeding session (years)	2.79 (1.54)	2.84 (1.65)	2.69 (1.27)	0.362
Unilateral or bilateral breast surgery				0.074
Unilateral	267 (88.1%)	179 (77.5%)	88 (86.3%)	
Bilateral	66 (11.9%)	52 (22.5%)	14 (13.7%)	
Diameter of the largest tumour (cm)				0.077
≤ 3 cm	290 (87.1%)	196 (84.8%)	94 (92.2%)	
>3 cm	43 (12.9%)	35 (15.2%)	8 (7.8%)	

‡ Data are presented as the mean ± SD and prevalence

* Indicates significantly different values at *p* < 0.05 (two-tailed)

### Breastfeeding of the first child born after surgery

**Fig. 1:**
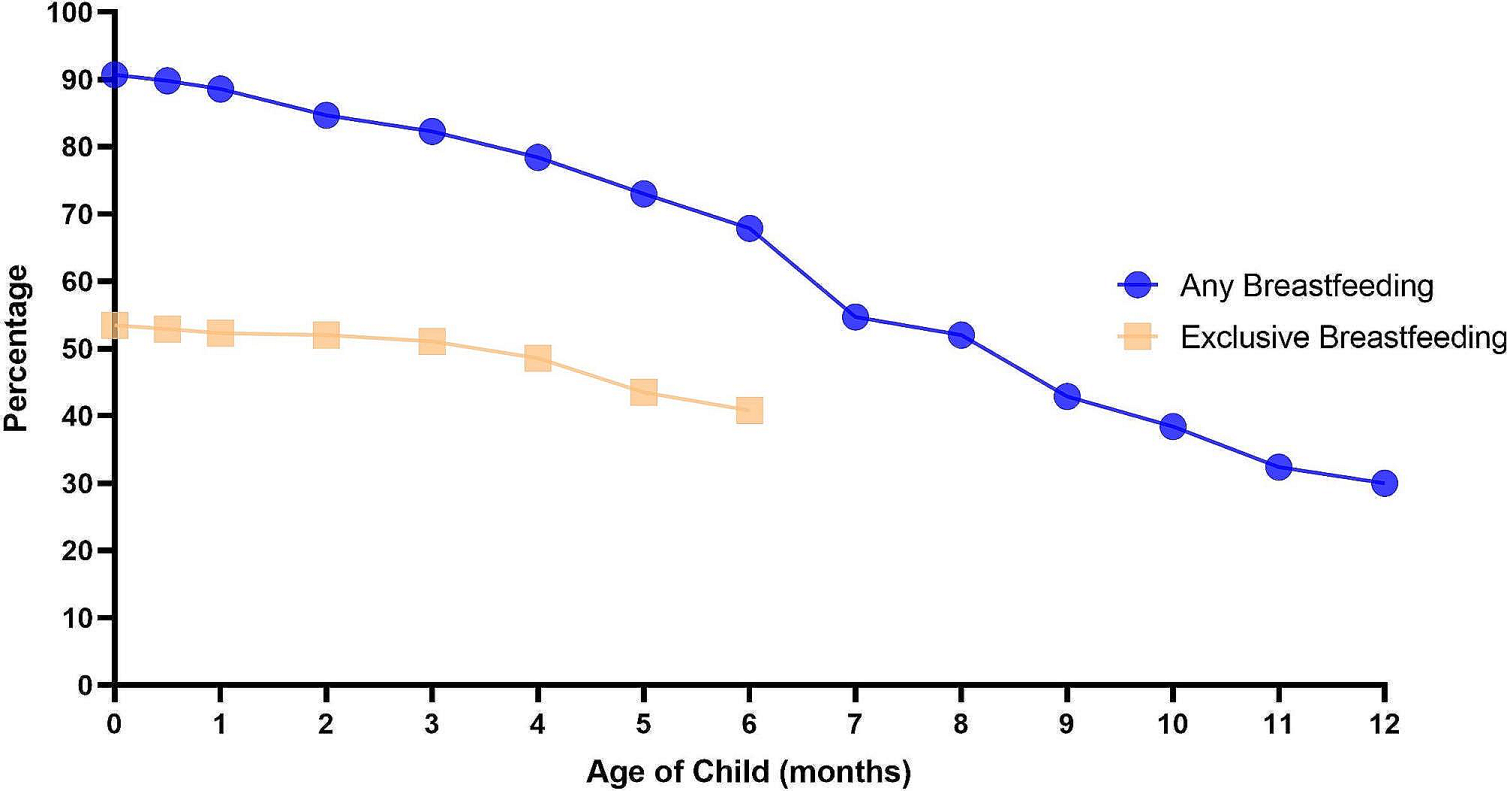
Rates of any and exclusive breastfeeding by child age among all patients enrolled

This study focused on breastfeeding of the first child born after surgery in 333 enrolled women who underwent surgery for benign breast disease. The rate of ever breastfeeding in this group was 91.0%, exclusive breastfeeding at six months was 40.8%, any breastfeeding at 12 months was 30.0% and any breastfeeding at 24 months was 3.3%. Excluding patients who did not breastfeed, the average duration of exclusive breastfeeding in this group was 5.1 months, and the average duration of any breastfeeding was 8.8 months (Table 3). The rates of exclusive breastfeeding and any breastfeeding decreased progressively over time among the 333 patients enrolled (Fig. 1).

**Table 3:** Breastfeeding outcomes for enrolled women

	Overall (333) ‡	Residents of first-tier cities (243) ‡	Residents of non-first tier cities (90) ‡	*P* value (t/U/χ2 test)	Patients with no history of childbirth prior to surgery (231) ‡	Patients with a history of childbirth prior to surgery (102) ‡	*P* value (t/U/χ2 test)
Average duration of any breastfeeding for the first child after surgery (months) †	8.80 (5.23)	8.68 (5.09)	9.09 (5.58)	0.711	8.75 (5.30)	8.91 (5.09)	0.799
Average duration of exclusive breastfeeding for the first child after surgery (months) †	5.08 (5.82)	4.69 (5.67)	6.07 (6.13)	0.044*	4.97 (5.84)	5.31 (5.81)	0.640
Whether ever breastfeeding				0.204			1.000
No	30 (9.0%)	25 (10.3%)	5 (5.6%)		21 (9.1%)	9 (8.8%)	
Yes	303 (91.0%)	218 (89.7%)	85 (94.4%)		210 (90.9%)	93 (91.2%)	
Exclusive breastfeeding duration for the first child after surgery				0.079			0.809
< 6 months	197 (59.2%)	151 (62.1%)	46 (51.1%)		138 (59.7%)	59 (57.8%)	
Any breastfeeding duration for the first child after surgery				0.423			0.437
< 12 months	233 (70.0%)	173 (71.2%)	60 (66.7%)		165 (71.4%)	68 (66.7%)	
> 12 months	100 (30.0%)	70 (28.8%)	30 (33.3%)		66 (28.6%)	34 (33.3%)	
Any breastfeeding duration for the first child after surgery				0.497			0.742
< 24 months	322 (96.7%)	236 (97.1%)	86 (95.6%)		224 (97.0%)	98 (96.1%)	
> 24 months	11 (3.3%)	7 (2.9%)	4 (4.4%)		7 (3.0%)	4 (3.9%)	

† Those who breastfed for less than 15 days in one month were considered to have a duration of 0 days; those who breastfed for more than 15 days but less than 1 month were considered to have a duration of 1 month

‡ Data are presented as the mean ± SD and prevalence

* Indicates significantly different values at *p* < 0.05 (two-tailed)

**Fig. 2:**
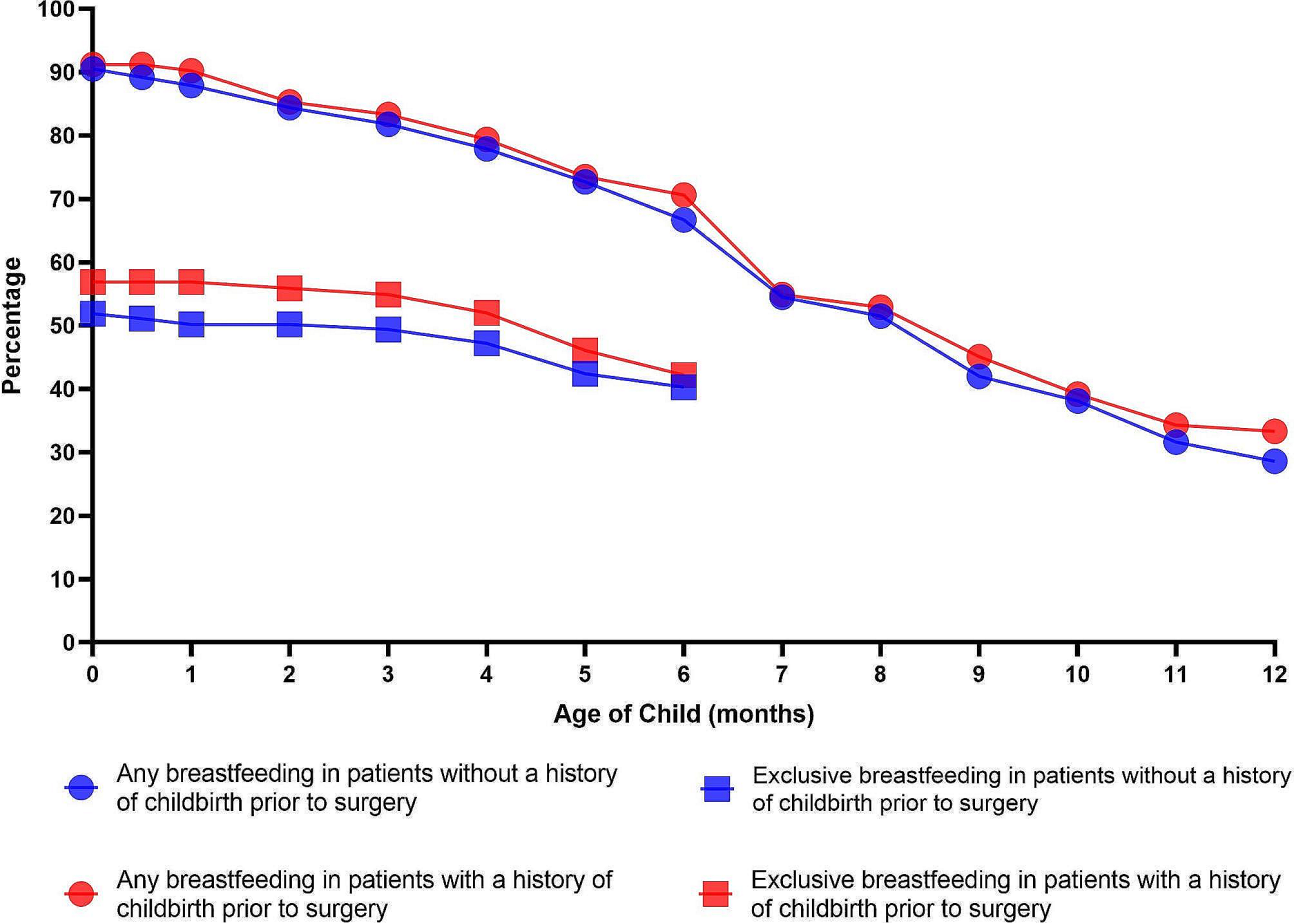
Rates of any and exclusive breastfeeding by child age among patients with or without a history of childbirth prior to surgery

Breastfeeding may also differ for the same woman at different births. We therefore regrouped the 333 enrolled patients into two groups: Group 1 included 231 patients with no history of childbirth prior to surgery and Group 2 included 102 patients with a history of childbirth prior to surgery. From Tables 2 and 3, we can see that the age, height, and weight of the patients with a history of childbirth prior to surgery were significantly greater than those of the patients without a history of childbirth prior to surgery. The number of tumours removed, average diameter of the largest tumour, and age at first birth of the patients with a history of childbirth prior to surgery were significantly lower than those of the patients without a history of childbirth prior to surgery. The rate of ever breastfeeding, the average duration of exclusive breastfeeding, the rate of exclusive breastfeeding at six months, and the rate of any breastfeeding at 12 and 24 months did not differ significantly between the two groups. The rates of exclusive breastfeeding and any breastfeeding in both groups decreased equally and progressively over time (Fig. 2).

We divided the 333 patients into two groups according to their place of residence. Group 1 consisted of 243 patients living in first-tier cities such as Guangzhou and Shenzhen, and Group 2 consisted of 90 patients living in non-first-tier cities (Table 4). The mean age of patients in the non-Tier 1 group was significantly younger than that of patients in the Tier 1 group. The mean diameter of the largest tumour in the non-Tier 1 group was significantly larger than that in the Tier 1 group, and the mother’s age at first birth in the non-Tier 1 group was significantly younger than that in the Tier 1 group.

**Table 4:** Baseline characteristics of enrolled patients grouped by place of residence

	Overall (333) ‡	Residents of first-tier cities (243) ‡	Residents of non-first tier cities (90) ‡	*P* value (t/U/χ2 test)
Age (years)	28.01 (4.11)	28.44 (4.02)	26.84 (4.14)	0.002*
Height (m)	1.59 (0.05)	1.59 (0.05)	1.58 (0.05)	0.223
Weight (kg)	50.08 (6.46)	50.43 (6.29)	49.13 (6.83)	0.102
BMI	19.88 (2.26)	19.98 (2.26)	19.62 (2.26)	0.201
Number of tumours removed	1.53 (0.95)	1.49 (0.92)	1.63 (1.00)	0.157
Diameter of the largest tumour (cm)	2.36 (1.04)	2.24 (1.00)	2.70 (1.09)	0.000*
Age at first birth (years)	28.73 (3.47)	28.97 (3.47)	28.10 (3.39)	0.042*
Interval between surgery and the first postoperative breastfeeding session (years)	2.79(1.54)	2.77 (1.56)	2.85 (1.49)	0.441
Education level				0.221
Junior college degree or above	288 (86.5%)	215 (88.5%)	73 (81.1%)	
Below junior college degree	34 (10.2%)	22 (9.1%)	12 (13.3%)	
Any history of childbirth prior to surgery				0.232
No	231 (69.4%)	164 (67.5%)	67 (74.4%)	
Yes	102 (30.6%)	79 (32.5%)	23 (25.6%)	
Unilateral or bilateral breast surgery				0.757
Unilateral	267 (88.1%)	196 (80.7%)	71 (78.9%)	
Bilateral	66 (11.9%)	47 (19.3%)	19 (21.1%)	
Diameter of the largest tumour (cm)				0.003*
≤ 3 cm	290 (87.1%)	220 (90.5%)	70 (77.8%)	
> 3 cm	43 (12.9%)	23 (9.5%)	20 (22.2%)	
Position of the incision				0.586
Margin of the areola	236 (70.9%)	169 (69.5%)	67 (74.4%)	
Tumour surface	80 (24.0%)	62 (25.5%)	18 (20.0%)	
Surgical method				0.129
Minimally invasive surgery	70 (21.0%)	57 (23.5%)	13 (14.4%)	
Conventional open surgery	261 (78.4%)	184 (75.7%)	77 (85.6%)	
Postoperative pathology				0.153
Fibroadenoma alone	253 (76.0%)	191 (78.6%)	62 (68.9%)	
Fibroadenoma with other benign lesions	29 (8.7%)	20 (8.2%)	9 (10.0%)	
Other benign disease	51 (15.3%)	32 (13.2%)	19 (21.1%)	
Redness, swelling and pain in the operated breast during breastfeeding				0.874
No	272 (81.7%)	199 (81.9%)	73 (81.1%)	
Yes	61 (18.3%)	44 (18.1%)	17 (18.9%)	

‡ Data are presented as the mean ± SD and prevalence

* Indicates significantly different values at *p* < 0.05 (two-tailed)

**Fig. 3:**
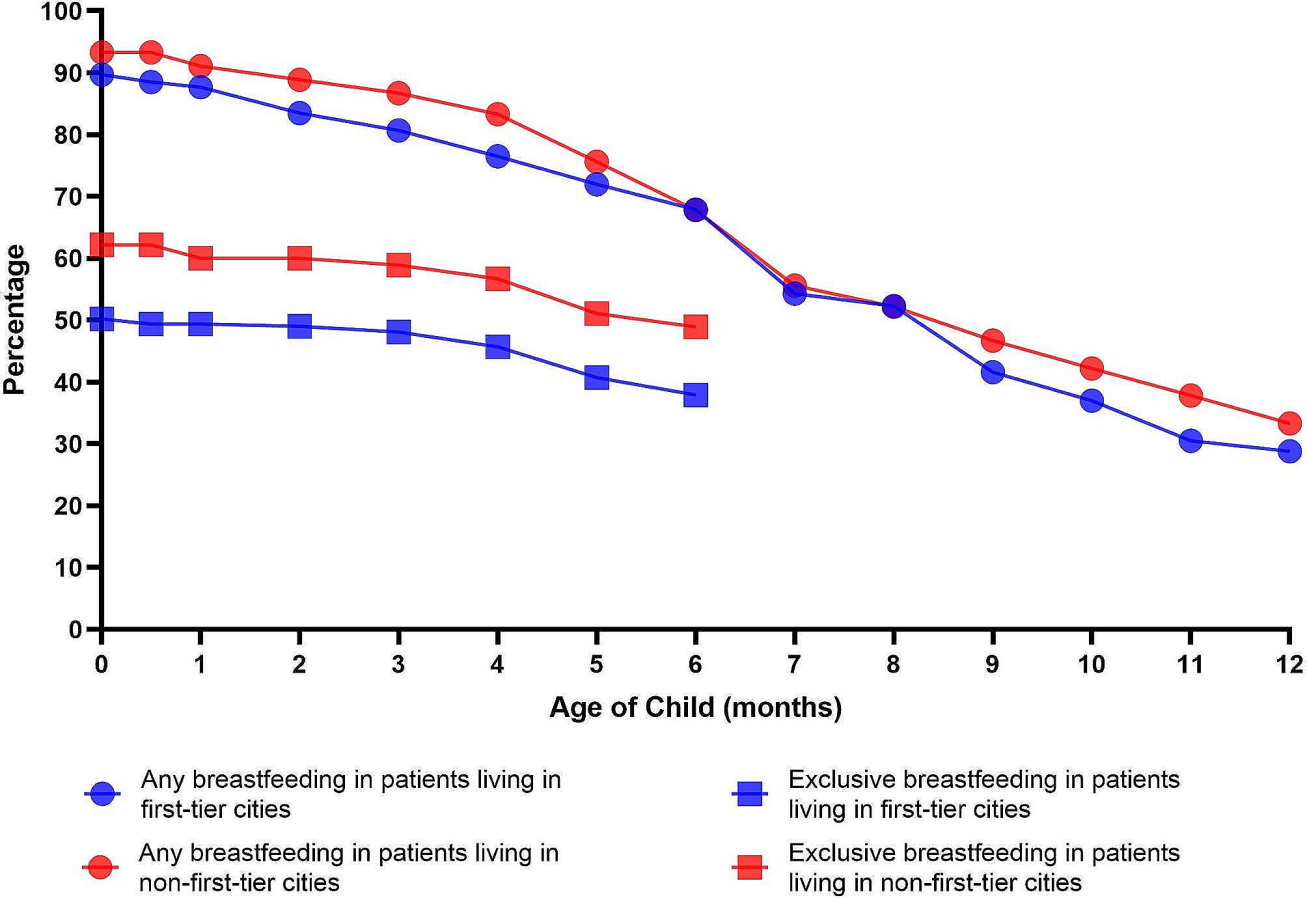
Rates of any and exclusive breastfeeding by child age among patients living in first-tier or non-first-tier cities

The mean duration of exclusive breastfeeding was significantly higher for patients in the non-Tier 1 group than for those in the Tier 1 group, but there was no significant difference in the rate of ever breastfeeding between the two groups. The mean duration of any breastfeeding was not significantly different between patients in the non-Tier 1 group and patients in the Tier 1 group. The rates of exclusive breastfeeding at six months and any breastfeeding at 12 and 24 months were higher for patients in the non-Tier 1 group than for patients in the Tier 1 group but were still not significantly different (Table 3). The rates of exclusive breastfeeding and any breastfeeding similarly decreased progressively with time in both groups (Fig. 3).

### Analysis of reasons for early breastfeeding cessation

Early cessation of breastfeeding (less than 15 days) occurred in 34 of the 333 patients enrolled. Of the 34 patients who stopped breastfeeding in the first 15 days, almost half cited insufficient milk as the reason for early cessation (Table 5).

**Table 5:** Reasons for early breastfeeding cessation (less than 15 days)

Reasons	Overall (34)	Residents of first-tier cities (28)	Residents of non-first tier cities (6)
Insufficient breast milk	15	13	2
Underlying diseases	3	2	1
Blocked breast ducts	2	1	1
Work	4	3	1
The child was hospitalised	1	1	0
Multiple reasons (e.g., insufficient breast milk, work, fatigue)	9	8	1

After excluding 34 patients who had early cessation of breastfeeding, a total of 163 patients exclusively breastfed for less than six months, of whom 42.9% stopped exclusive breastfeeding because of work, 34.3% stopped because of insufficient breast milk and 22.6% stopped for multiple reasons (e.g., insufficient breast milk, work, early addition of complementary foods) (Table 6); in addition, a total of 203 patients breastfed for less than 12 months.

**Table 6:** Reasons for exclusive breastfeeding for less than 6 months

Reasons	Overall (163)	Residents of first-tier cities (123)	Residents of non-first tier cities (40)
Work	70	50	20
Insufficient breast milk	56	45	11
Multiple reasons (e.g., insufficient breast milk, work, early addition of complementary foods)	37	28	9

### Psychological impact of surgery for benign breast diseases on this group of women

Through this questionnaire, we found that of the 267 patients who underwent surgery for unilateral benign breast disease, 96 patients perceived that they were breastfeeding significantly less on the operated breast than on the healthy breast. In addition, of the 303 patients who had ever breastfed after surgery, 88 (29.0%) voluntarily reduced the frequency and duration of breastfeeding on the operated breast because of the surgery.

## Discussion

To our knowledge, our study is the first investigation describing breastfeeding in women of childbearing age after undergoing surgery for benign breast disease. In our study, after undergoing surgery for BBD, the rate of ever breastfeeding for this group of patients was 91.0%, which was slightly lower than the national average of 93.7% [12]; the rate of any breastfeeding at 12 months was 30.0%, which was well below the national average of 66.5%. The six-month exclusive breastfeeding rate for these patients was 40.8%, which seems higher than the national average of 29.2% [12] but still below the 50% WHO recommendation. In addition, the first-tier cities are significantly better than the non-first-tier cities in China in many aspects, such as economy, education, health care, and public facilities. However, our investigation found that the mean duration of exclusive breastfeeding for the first child born after surgery was significantly higher in patients living in non-first-tier cities than in those living in first-tier cities (6.07 vs. 4.69 months, *P* = 0.044). Moreover, the rates of exclusive breastfeeding at six months and any breastfeeding at 12 and 24 months were higher among patients living in non-first-tier cities than among those living in first-tier cities, which was likely due to the higher work pressure.

Many studies have indicated that insufficient breastmilk is the most common reason for stopping breastfeeding [19, 21]. Similarly, our study found that the majority of women who had undergone surgery for benign breast disease reported that their early cessation of breastfeeding was due to insufficient breastmilk, and a small proportion had early cessation of breastfeeding due to underlying diseases, blockage of the milk ducts, work and fatigue. Most of the patients who stopped exclusive breastfeeding within six months stated that it was due to work, followed by insufficient breastmilk, and most of the patients who stopped breastfeeding within 12 months also stopped due to work, followed by insufficient breast milk, and the baby’s refusal to suck. There was no significant difference in the reasons for breastfeeding cessation between patients in first-tier cities and those in non-first-tier cities. Work can affect breastfeeding in several ways. From the line graph of breastfeeding status, we can see that the curve for any breastfeeding declines the most after six months, which may be related to the paid maternity leave system [22]. In the Pearl River Delta region of Guangdong Province, China, the duration of paid maternity leave in most establishments ranges from three to six months, with six months of paid maternity leave being more common, so that patients have enough time for rest and breastfeeding. After the six-month paid maternity leave, busy schedules make many patients choose to stop exclusive breastfeeding or to stop breastfeeding completely. In addition, most of the institutions in China lack special breastfeeding rooms and do not provide special time for breastfeeding, so women who return to work encounter many difficulties if they wish to continue breastfeeding.

Moreover, there are many different brands of infant formula currently available in the community. To pursue profits, businesses produce advertisements, and some of the advertisements go so far as to falsely exaggerate the benefits of infant formula, to profit by deception [19]. Studies have shown that baby food marketing undermines breastfeeding by favourably influencing women’s attitudes and decision-making towards commercial baby food. Women who experienced baby food marketing at health facilities were four times more likely to feed commercial milk formula (CMF) to their children than those who did not experience such marketing [23].

There is a very close interrelationship between breastfeeding and BBDs. In young women, breastfeeding and pregnancy are protective against malignant breast disease but are likely to promote the development of BBD due to hormonal changes [24, 25]. Studies have shown that there is no significant difference in breastfeeding among women with different types of BBDs, but the duration of lactation is significantly correlated with the number of fibroadenomas [5]. The main treatment for benign breast disease is surgery, which can relieve the pressure of the tumour on the milk ducts, but at the same time, surgery may also damage the milk ducts, which may lead to a reduced or inability to breastfeed. There have been a handful of studies exploring the intrinsic relationship between breast surgery and breastfeeding, but no definitive answer has been reached yet [26].

Research has shown that lactation seems to depend on the presence of intact lobules in the breast, intact lactiferous ducts, and an intact nerve supply to the nipple to respond to the sucking reflex [27]. The impact of surgery on breastfeeding depends on the type of intervention and surgical technique [28]. Lactation is altered after either reduction or augmentation breast surgery [14, 26, 29]. In addition, the type of breast incision is significantly associated with lactation outcomes [29]. For cosmetic reasons, the majority of surgical incisions for benign breast disease are now made at the areola margin, which may increase the risk of damaging the nerves around the nipple areola, resulting in altered nipple sensitivity and thus affecting the reflex arc, which is normally stimulated by sucking. It has been shown that periareolar incision and nipple hypoesthesia after surgery are likely to result in diminished lactation [28]. However, techniques that preserve the column of subareolar parenchyma appear to lead to a greater likelihood of breastfeeding [14]. Consequently, the surgical technique, location of the incision, distance between the tumour and nipple, amount of glandular tissue removed and resultant nipple sensitivity have been considered to be important factors influencing lactation [27].

Studies have shown that many women report either not trying to breastfeed or failing to breastfeed after breast surgery. The primary reason for not being able to breastfeed was insufficient milk production, while the main reason for not trying to breastfeed involved psychosocial factors, including personal discouragement, dissuasion by health professionals and occupational demands [30–34]. Our study found that 29% of the enrolled patients reported that they had previously voluntarily reduced the frequency and duration of breastfeeding on the operated breast because of the surgery. Surgery can cause some damage to the structural integrity of the breast, but whether this structural change affects breastfeeding function is not known at this time. However, we can see from the questionnaire that the surgery did have some negative impact on the patients’ psychology regarding breastfeeding. Therefore, we must focus on providing more breastfeeding education to women of childbearing age who have undergone breast surgery, regardless of whether they have undergone surgery for benign breast diseases or malignant tumours, and society and families should also provide more support and encouragement. Next, we will recruit women of childbearing age who have undergone mammary lumpectomy and those who have not, and prospectively observe and analyse the effects of such surgery on breastfeeding and associated risk factors.

### Strengths and limitations

The greatest strength of our retrospective survey study is that it is the first to describe breastfeeding in women of childbearing age after BBD surgery and to further analyse the reasons for breastfeeding cessation in this population. Although our study could not prove whether BBD surgery affects breastfeeding function, the survey data showed that the average duration of breastfeeding after surgery for this group of women is not worse than the national average. This could help to reduce concerns about surgery in young women with benign breast disease. Our findings could greatly encourage these patients to breastfeed actively after surgery, reduce their psychosomatic stress, increase their self-confidence in breastfeeding, and prolong the duration of postoperative breastfeeding.

The limitations of our study include the following: first, the number of patients enrolled was not large enough; second, as it was a retrospective study, patients were asked to recall the duration of breastfeeding, so the data may be somewhat biased; third, our study only included patients who underwent surgery for benign breast disease and finally, we did not have access to patient information from other studies, therefore we were not able to add statistics for control variables. Further prospective clinical studies are still needed if we want to investigate whether surgery for benign breast disease affects breastfeeding.

## Conclusions

In conclusion, our survey found that there are some impacts of benign breast disease surgery on breastfeeding, some of which may be psychological. We found significantly lower rates of ever breastfeeding and any breastfeeding at 12 months among women of childbearing age after undergoing BBD surgery than the national average in China. The rate of exclusive breastfeeding at six months among these patients, although significantly higher than the average, still fell short of the WHO recommended 50% standard. We must focus on providing more breastfeeding education to women of childbearing age who have undergone surgery for benign breast disease, especially to emphasise the importance of continued breastfeeding after six months. In addition, institutions should provide more facilities for mothers who have undergone breast surgery to help them breastfeed, such as conducting community education on breastfeeding after breast surgery, training professional postoperative lactation consultants in hospitals, extending maternity leave, and providing private breastfeeding rooms on the work place. Families should also provide adequate support and encourage mothers to breastfeed with both breasts instead of only the non-operated breast.

## Data Availability

The questionnaire and datasets used during the current study are available from the corresponding author upon reasonable request.

## Abbreviations

BBD: Benign breast disease
SD: Standard deviation
BMI: Body mass index
WHO: World Health Organization
CDC: Centers for Disease Control and Prevention
CMF: Commercial milk formula
UNICEF: United Nations International Children’s Emergency Fund
